# Single-Stage Off-Pump Hybrid Repair of Kommerell's Diverticulum with Right-Sided Aortic Arch Using a Surgeon-Customized Vascular Prosthesis

**DOI:** 10.1055/s-0041-1732397

**Published:** 2021-12-08

**Authors:** Metesh Acharya, Aamer Ahmed, Aparna Deshpande, Tryfon Vainas, Leonidas Hadjinikolaou, Giovanni Mariscalco

**Affiliations:** 1 Department of Cardiac Surgery, Glenfield Hospital, Leicester, United Kingdom; 2 Department of Anaesthesia, Glenfield Hospital, Leicester, United Kingdom; 3 Department of Radiology, Glenfield Hospital, Leicester, United Kingdom; 4 Department of Vascular Surgery, Glenfield Hospital, Leicester, United Kingdom

**Keywords:** Kommerell's diverticulum, right aortic arch, aortic aneurysm, hybrid approach, thoracic endovascular aortic repair

## Abstract

We report the successful single-stage hybrid management of Kommerell's diverticulum associated with a right-sided aortic arch in a 63-year-old woman. She underwent total aortic arch debranching utilizing a surgeon-customized vascular prosthesis, without cardiopulmonary bypass or deep hypothermic circulatory arrest, and concomitant zone-0 endovascular stent–graft deployment.

## Introduction

The management of Kommerell's diverticulum (KD), an aneurysmal dilatation of the aorta at the origin of an aberrant right subclavian artery in a left-sided aortic arch (type I) or aberrant left subclavian artery in a right-sided aortic arch (type II), has traditionally involved an open two-stage elephant trunk procedure. In the last two decades, however, advancements in thoracic endovascular aortic repair (TEVAR) have stimulated interest in less invasive hybrid approaches in the treatment of complex aortic arch aneurysms and dissections.

We, here, report the single-stage hybrid repair for a type-I KD using a surgeon-customized vascular prosthesis for total aortic arch debranching without cardiopulmonary bypass (CPB) or hypothermic circulatory arrest (HCA), alongside concomitant endovascular stent–graft deployment.

## Case Presentation

**Fig. 1:**
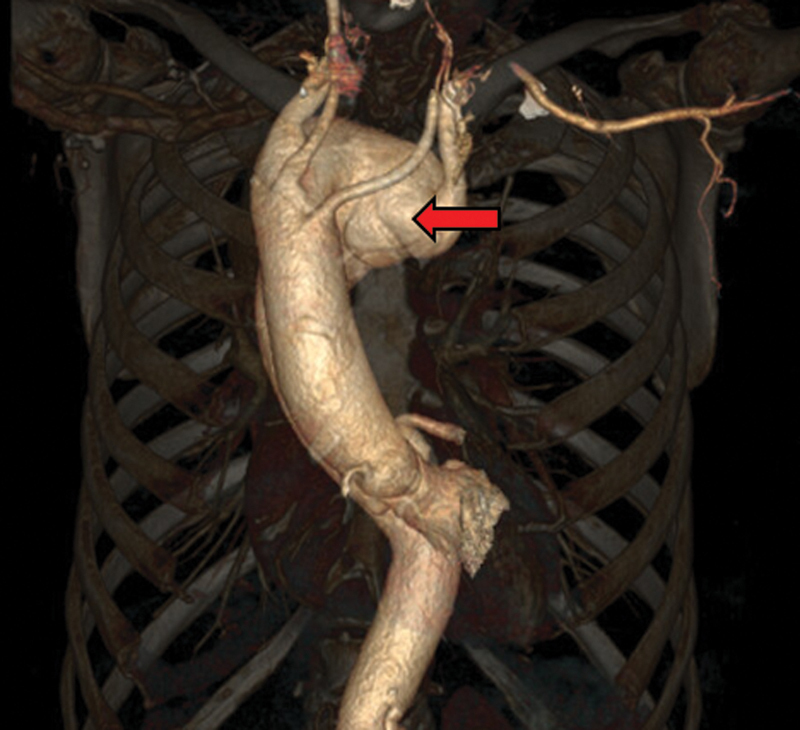
Reformatted preoperative three-dimensional computed tomography scan demonstrating a Kommerell's diverticulum (red arrow) associated with a right-sided aortic arch.

A 63-year-old frail woman with hypertension and smoking history presented for investigation of dysphagia associated with moderate dyspnea and chest pain. Esophagogastroduodenoscopy was normal. Computed tomography (CT) scanning ( Fig. 1 ) revealed a right-sided aortic arch with a complex anomalous configuration, comprising a 9.5 cm × 5.5 cm KD displacing the esophagus anteriorly, an aberrant left common carotid artery originating from the ascending aorta as a first branch, followed by the right common carotid and right subclavian arteries as second and third branches. The maximal diameters of the ascending aorta and descending aorta were 3.7 and 3.2 cm, respectively. Transthoracic echocardiography demonstrated preserved biventricular function and pulmonary function tests showed a mild restrictive defect.

Following multidisciplinary panel consensus, we planned a hybrid strategy in this high-risk patient, consisting of off-pump aortic arch debranching to isolate the KD, while preserving cerebral perfusion and avoiding the deleterious effects of prolonged CPB and HCA. This would enable a suitable proximal landing zone for concomitant TEVAR, intended to avoid the significant morbidity and mortality risks associated with prospective open thoracic intervention for an aneurysmal descending aorta.

Surgery was performed in a dedicated hybrid operating theater. Following median sternotomy, the pericardial sac was opened, and the ascending aorta, aortic arch, neck vessels, and axillary arteries were exposed after full-dose systemic heparinization. A 34-mm diameter Gelweave Lupiae pentafurcate Dacron prosthesis (Vascutek Ltd., Glasgow, Scotland) was modified by trimming off its trifurcated portion, consisting of three branches measuring 10, 10, and 8 mm in diameter, the proximal “inflow” end of which was anastomosed to the anterolateral aspect of ascending aorta over a side-biting clamp.

**Fig. 2:**
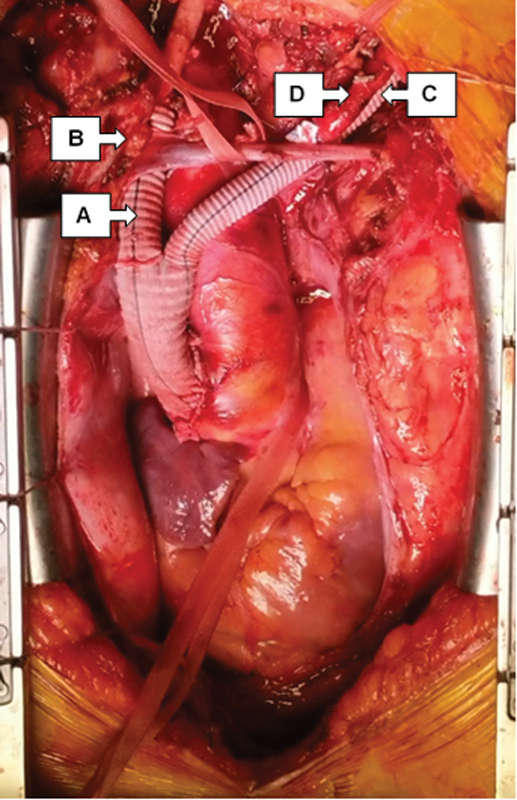
Intraoperative photograph showing customized vascular graft for aortic debranching with proximal end-to-side anastomosis to the native ascending aorta. **A** , graft to right common carotid artery; **B** , graft to right axillary artery (under **A** ); **C** , graft to left subclavian artery; and **D** , left common carotid artery transposed onto **C** .

The right axillary, right common carotid, and left subclavian arteries were sequentially anastomosed in end-to-end fashion with the branches of the precustomized trifurcate prosthesis ( Fig. 2 ). The supra-aortic vessels were disconnected from the aortic arch and their origins were occluded with pledgeted polypropylene sutures and vascular clips.

A left carotid-subclavian transposition was then performed, by detaching the left common carotid artery from the aortic arch and end-to-side anastomosis to the left subclavian artery graft.

Finally, an endovascular stent–graft (Relay NBS, Terumo Aortic, Renfrewshire, Scotland) was deployed retrogradely through the right common femoral artery for descending aortic reconstruction extending proximally into zone 0. Completion aortography revealed patent debranching grafts and no endoleak.

**Fig. 3:**
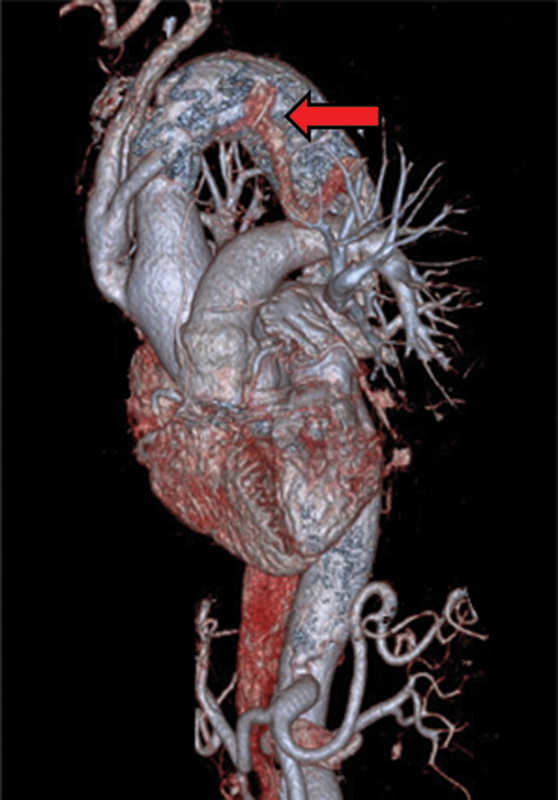
Reformatted postoperative three-dimensional computed tomography scan showing the debranched aorta and endovascular stent graft (red arrow).

Postoperatively, the patient required permanent pacemaker insertion for complete atrioventricular block. She was discharged home in a good functional state with no neurological deficit. Follow-up CT angiography ( Fig. 3 ) at 2.5 months confirmed a satisfactory aortic repair with good supra-aortic graft patency and stable stent–graft position.

## Discussion

KD is a rare congenital abnormality characterized by aneurysmal dilatation of the origin of an aberrant subclavian artery. It may be clinically silent or manifest with symptoms from compression of neighboring mediastinal structures including chest pain, dyspnea, or dysphagia. With the risk of rupture or dissection in KD reported at 19 to 53%, 1 prophylactic repair has been advocated with a diameter exceeding 3 to 5 cm. 2 3 While KD is a rare entity, greater insight into its natural history and analysis of complication rates has recently led to the development of a management algorithm by the Yale group. 4

Staged open surgical repair has traditionally represented the favored approach for KD repair, employing HCA or partial left heart bypass via median sternotomy and thoracotomy for total arch replacement using an interposition graft. 3 The associated perioperative mortality was reported at 40% in an early report by Austin and Wolfe, 5 although Kouchoukos and Masetti 3 observed no deaths in a more contemporary series utilizing left thoracotomy for graft replacement as a standardized open approach.

Accruing experience with TEVAR has facilitated the adoption of sophisticated and less-invasive hybrid strategies for the management of complex arch aneurysms and dissections with encouraging results, aiming to mitigate operative trauma through avoidance of a thoracotomy. Hybrid techniques include two-stage classical elephant trunk repair, single-stage frozen elephant trunk procedure, partial or total arch debranching, or coil embolization of the KD with subclavian artery revascularization, all combined with completion TEVAR. 1 Sternotomy additionally permits antegrade stent–graft delivery via the ascending aorta to restrict iliofemoral access. Recognized complications of TEVAR include endoleak, aortooesophageal fistula formation, arm claudication, and retrograde dissection.

Total endovascular repair has been proposed as a feasible technique in high-risk patients or emergent cases characterized by rupture but is dependent on adequate proximal landing zones. 6 Furthermore, the negotiation of challenging anatomy necessitates expert endovascular skills which limits its wider application. 1

The choice of procedure for KD repair essentially depends on patient comorbidity, arch and supra-aortic vessel anatomy, and surgical experience. In our frail patient with poor functional reserve who was at high risk of perioperative complications, we elected for off-pump aortic arch debranching with concomitant TEVAR.

This single-stage hybrid procedure confers several advantages, particularly in those patients with multiple comorbidities in whom the morbidity burden of extensive open surgery would be prohibitive. First, it simultaneously achieves exclusion of the KD from the systemic circulation and revascularization of the supra-aortic vessels while maintaining cerebral perfusion under normothermic conditions. We specifically wished to avoid HCA and its attendant risks of coagulopathy, neurological injury, and renal and respiratory failure. Left subclavian artery revascularization is important for spinal cord protection. Second, this technique prophylactically addresses the more distal aorta through endovascular stent–graft placement, thereby hopefully obviating the need for challenging open thoracoabdominal intervention in the future, making it an attractive option in Type B acute aortic syndromes. As an additional benefit, this approach avoids full aortic cross-clamping and CPB, since anastomosis of the modified trifurcated vascular graft to the ascending aorta requires only a short duration of partial clamping. Partial aortic clamping was deemed safe in our patient as the native ascending aorta was not sufficiently dilated to warrant replacement or a reduction procedure, and thus presented a satisfactory proximal landing zone of nonaneurysmal aorta to accommodate a stent graft.

Dysphagia in patients with KD may be attributed to a vascular ring phenomenon causing extrinsic esophageal compression. We anticipated that transection of the supra-aortic vessels in combination with the resultant decompression of the large arch aneurysm would relieve any significant pressure on the esophagus, without necessitating an accompanying procedure for interruption of the vascular ring. Indeed, our patient's dysphagia gradually resolved postoperatively.

In conclusion, this case demonstrates the successful single-stage hybrid management of KD associated with complex aortic arch pathology without CPB or HCA. We suggest that this reproducible approach is particularly applicable in higher risk patients whose comorbidity profile may otherwise preclude conventional open repair.
